# The co-presence of deletion 7q, 20q and inversion 16 in therapy-related acute myeloid leukemia developed secondary to treatment of breast cancer with cyclophosphamide, doxorubicin, and radiotherapy: a case report

**DOI:** 10.1186/1752-1947-6-67

**Published:** 2012-02-16

**Authors:** Ipek Yonal, Fehmi Hindilerden, Erkan Ozcan, Sukru Palanduz, Melih Aktan

**Affiliations:** 1 Istanbul University Istanbul Medical Faculty, Department of Internal Medicine, Division of Hematology, Istanbul, Turkey; 2 Istanbul Bilim University, Department of Internal Medicine, Division of Hematology, Istanbul, Turkey; 3 Istanbul University Istanbul Medical Faculty, Department of Internal Medicine, Division of Medical Genetics, Istanbul, Turkey

## Abstract

**Introduction:**

Therapy-related acute myeloid leukemia occurs as a complication of treatment with chemotherapy, radiotherapy, immunosuppressive agents or exposure to environmental carcinogens.

**Case presentation:**

We report a case of therapy-related acute myeloid leukemia in a 37-year-old Turkish woman in complete remission from breast cancer. Our patient presented to our facility with fatigue, fever, sore throat, peripheral lymphadenopathy, and moderate hepatosplenomegaly. On peripheral blood and bone marrow aspirate smears, monoblasts were present. Immunophenotypic analysis of the bone marrow showed expression of CD11b, CD13, CD14, CD15, CD33, CD34, CD45 and human leukocyte antigen-DR, findings compatible with the diagnosis of acute monoblastic leukemia (French-American-British classification M5a). Therapy-related acute myeloid leukemia developed three years after adjuvant chemotherapy consisting of an alkylating agent, cyclophosphamide and DNA topoisomerase II inhibitor, doxorubicin and adjuvant radiotherapy. Cytogenetic analysis revealed a 46, XX, deletion 7 (q22q34), deletion 20 (q11.2q13.1) karyotype in five out of 20 metaphases and inversion 16 was detected by fluorescence in situhybridization. There was no response to chemotherapy (cytarabine and idarubicin, FLAG-IDA protocol, azacitidine) and our patient died in the 11th month after diagnosis.

**Conclusions:**

The median survival in therapy-related acute myeloid leukemia is shorter compared to de novoacute myeloid leukemia. Also, the response to therapy is poor. In therapy-related acute myeloid leukemia, complex karyotypes have been associated with abnormalities of chromosome 5, rather than 7. To the best of our knowledge, this is the first case of therapy-related acute myeloid leukemia showing the co-presence of deletion 7q, 20q and the inversion 16 signal.

## Introduction

Therapy-related acute myeloid leukemias (t-AML) are long-term adverse consequences in malignant diseases treated with chemotherapy, radiation therapy or immunosuppressive agents [1]. The commonest subtype of t-AML develops after exposure to alkylating agents. Most cases present with monosomy/deletion of chromosome 5q and/or monosomy/deletion of 7q [2,3]. The majority of alkylating agents and radiotherapy treatments damage DNA by methylation [1]. Balanced translocations involving chromosome bands 11q23 and 21q22 may develop following therapy with DNA topoisomerase II inhibitors [4]. Inversion 16 (inv(16)) is one of the less frequent genetic alterations observed after exposure to DNA topoisomerase II inhibitors [4]. In the Chicago University series of 386 patients with therapy-related myelodysplastic syndrome (t-MDS) and t-AML, only eight (2%) patients had abnormalities of -Y, +11, del(11q), del(20q), +21 [5]. Qian et al*. *reported that complex karyotypes in primary MDS, AML de novo, or t-MDS/t-AML were associated with abnormalities of chromosome 5, rather than chromosome 7 [5]. Here, we report a case of t-AML developed after adjuvant chemoradiotherapy for breast cancer. Cytogenetic analysis revealed abnormalities of both chromosomes 7 and 20 (del(7q) and del(20q)). Fluorescence in situ hybridization (FISH) analysis revealed the inv(16) signal.

## Case presentation

**Figure 1:**
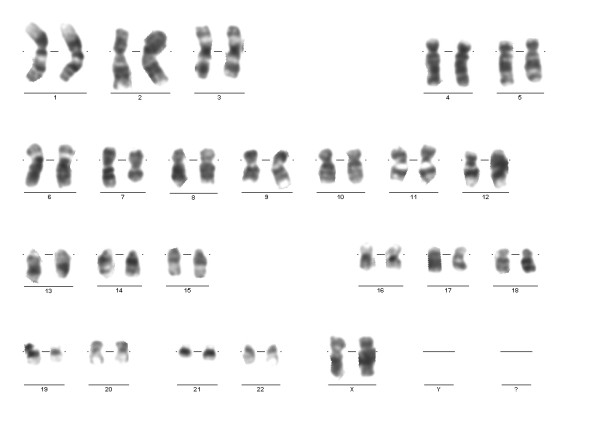
**Karyogram showing deletion 7q and deletion 20q**.

A 37-year-old Turkish woman was examined for a mass in the upper lateral quadrant of her right breast three years ago. She had a family history of endometrial carcinoma. A mammography study revealed a 22 mm nodular lesion with irregular peripheral borders in the lateral upper quadrant of her right breast. There were no palpable axillary lymph nodes. A 3.2 × 2.2 × 2 cm mass in the right breast was detected on breast ultrasonography (USG). An incisional biopsy of the mass led to a diagnosis of invasive ductal carcinoma and a breast-conserving partial mastectomy of the right breast with axillary dissection was performed. There were no metastases detected on abdominal USG, thorax tomography and bone scintigraphy. Pathological examination of the operation material revealed invasive ductal carcinoma. The tumor was found to be stage IIA (T2N0M0). Four cycles of AC combination chemotherapy (adriamycin 40 mg/m^2 ^on day one and cyclophosphamide 200 mg/m^2^/day on days three to six at 28-day intervals) was given. In addition, adjuvant radiotherapy (60.4 Gy for 33 days) was performed. There was no metastasis or recurrence detected in the period during and after adjuvant treatment. Three years after chemotherapy, our patient was admitted to our emergency department because of fatigue, fever and sore throat. On clinical examination, hepatosplenomegaly (both 2 cm below the costal margins), cervical lymphadenopathy and petechiae were noted. Her laboratory test results were as follows: hemoglobin 9 g/dL, hematocrit 32%, total leukocytes 343,000 cells/mm^3^, platelets 79,000 cells/mm^3^, erythrocyte sedimentation rate 51 mm/hour, lactate dehydrogenase (LDH) 2250 U/L, creatinine 1.3 mg/dL, uric acid 5.5 mg/dL, calcium 8.8 mg/dL, phosphorus 3.5 mg/dL and C-reactive protein 21.1 g/L. A peripheral blood smear showed diffuse myeloblasts. Antibacterial therapy was started, with a diagnosis of febrile neutropenia. Our patient was referred to our hematology department. A bone marrow aspirate revealed a 100% infiltration composed of monoblasts. A subsequent bone marrow biopsy showed diffuse infiltration by leukemic blast cells that were diffusely positive for CD34, and focally positive for CD33, myeloperoxidase and lysozyme. Immunophenotypic analysis of the bone marrow was positive for CD11b, CD13, CD14, CD15, CD33, CD34, CD45 and human leukocyte antigen (HLA)-DR. Based on these data, a diagnosis of acute monoblastic leukemia (French-American-British (FAB) classification M5a) was made. On cytogenetic analysis of the bone marrow, five of the examined 20 metaphases revealed a 46, XX, del(7)(q22q34), del(20)(q11.2q13.1) karyotype (Figure 1). Karyotypes were coded according to International Standing Committee on Human Cytogenetic Nomenclature (ICSN) guidelines [6]. FISH analysis of the bone marrow revealed an inv(16) signal. A remission induction regime including cytarabine 100 mg/m^2 ^daily on days one to seven and idarubicin 12 mg/m^2 ^daily on days one to three was initiated. On the 28th day of the regimen, no remission was obtained. Therefore, re-induction therapy with the same regimen was given. On the 28th day of the re-induction therapy, bone marrow aspirate revealed 85% monoblasts. Because of refractory disease, FLAG-IDA chemotherapy, a more aggressive regimen consisting of a five-day course of 30 mg/m^2 ^per day fludarabine, 2 g/m^2 ^per day cytosine arabinoside, granulocyte colony-stimulating factor, and a three-day course of idarubicin 8 mg/m^2 ^was applied. After 10 days of persistent fever despite broad-spectrum antibacterial therapies, empirical antifungal treatment was added. Thorax tomography revealed bilateral diffuse ground-glass opacities and bilateral pleural effusions. Serum galactomannan was positive. The final diagnosis was invasive fungal aspergillosis of the lung. On the 28th day of the FLAG-IDA protocol, bone marrow aspirate revealed 80% monoblasts and on cytogenetic analysis of the bone marrow; five of the examined 20 metaphases still revealed a 46, XX, del(7)(q22q34), del(20)(q11.2q13.1) karyotype. The inv(16) signal disappeared on FISH analysis. One schedule of azacitidine (75 mg/m^2^/day subcutaneously for seven days) and two schedules of azacitidine (100 mg/m^2^/day subcutaneously for seven days) every 28 days were performed. However, no remission was obtained and our patient died 10 months after diagnosis due to sepsis and pulmonary failure.

## Conclusions

The World Health Organization (WHO) classification of therapy-related myeloid neoplasms (t-MN) includes t-AML, t-MDS, or myelodysplastic syndrome/myeloproliferative neoplasms (t-MDS/MPN) that develop secondary to exposure to cytotoxic agents or radiation [7]. The median survival is shorter compared to de novoAML, MDS, or MDS/MPN. Also, the response to therapy is poor. In our patient's case, remission was never achieved. The latency period between first exposure to a cytotoxic agent and the development of t-MN ranges from one to 10 years. t-MN occurs five to seven years after exposure to alkylating agents and radiotherapy [8,9]. This period was one to three years after the use of DNA topoisomerase II inhibitors [10,11]. Our patient presented to our facility in a leukemic state three years after exposure to chemoradiotherapy.

t-MN has been reported after chemotherapy with alkylating agents and/or topoisomerase inhibitors for the treatment of Hodgkin's disease, non-Hodgkin's lymphoma, acute lymphoblastic leukemia, breast cancer, ovarian carcinoma, or testicular carcinoma [12-16]. Among solid tumors, breast cancer is the one most commonly associated with an increased risk of development of t-MN.

Taking into consideration the increase in survival and overall cure rate of patients with cancer, the potential risk of therapy-associated secondary malignancies is of growing interest. Nowadays, this issue is becoming particularly important as a significant number of patients with breast cancer receive adjuvant chemotherapy following complete surgical resection. Adjuvant chemotherapy improves survival among women with high-risk breast cancers [17]. The late side effects related to chemotherapy in this particular group of patients need to be clearly elucidated. Certain cytotoxic drugs increase the risk of developing a secondary malignancy. Specifically, t-MN can develop after treatment with alkylating agents and topoisomerase II reactive drugs. Treatment for breast cancer is associated with a 1% to 5% lifetime risk of t-MN. Praga et al*. *analyzed 9796 patients with breast cancer treated in 19 randomized trials. The cumulative eight-year risk of t-AML was reported as 0.55% [18]. The risk of t-MN increased with higher cumulative doses of both alkylating agents and anthracyclines [18]. Martin et al*. *reported that younger age at the time of diagnosis, advanced stage disease with distal involvement and radiotherapy were significant risk factors for t-MN in patients with breast cancer [19]. In our patient, who received standard-dose chemotherapy, the identified risk factors for t-AML were young age at the time of diagnosis and radiotherapy.

Alkylating agents have been shown to affect tissue DNA methylation profile [1]. Radiation is also able to induce a stable DNA hypomethylation in both target and bystander tissues. t-MN following ionizing radiations can be assimilated to leukemias following alkylating agents. Voso et al*. *demonstrated the correlation between p15 methylation and monosomy/deletion of chromosome 7q, proposing it as a relevant event in alkylating-agent-induced leukemogenesis [1]. In alkylating-agent-induced t-MN, 60% to 90% of patients present with monosomy/deletion of 5q and/or monosomy/deletion of 7q [4]. The first subgroup includes cases of t-MN with monosomy/deletion of 5q, with or without abnormalities of chromosome 7. Such cases often present monosomy of chromosome 17 or deletion or loss of 17p, duplication or amplification of chromosome band 11q23, and a complex karyotype with many unidentified abnormalities. The second subgroup of t-MN includes cases with monosomy/deletion of 7q, but normal chromosome 5. Qian et al*. *reported cytogenetic pattern of 3444 patients with primary MDS, AML de novo, or t-MN. They found that 16% of patients had del(5q), and 17.3% had del(7q). Complex karyotypes were associated with abnormalities of chromosome 5, rather than 7. Recurring abnormalities observed at a high frequency (> 20%) in patients with del(5q) included +8, and loss of 13q, 16q, 17p (40% of cases), chromosome 18, and 20q [5]. In our patient, however, other genetic abnormalities (del 20q and the inv(16)) were present in addition to the chromosomal abnormality on chromosome 7.

The latency between the alkylating agents and t-MN is between five and seven years. This group often exhibits a M1 or M2 phenotype (FAB classification). Generally, overt leukemia develops following a dysplastic phase. t-MN related to alkylating agents typically has a poor prognosis [4].

Chromosomal breakage induced by DNA topoisomerase II inhibitors is responsible for its antineoplastic and leukemogenic effects, and this breakage resolved by chromosomal translocation is the cause of the leukemic transformation [4]. t-MN after DNA topoisomerase II inhibitors typically has a short latency time, ranging between one and three years from primary treatment [4,15]. Among the genetic derangements associated with DNA topoisomerase II inhibitors, translocations at the long arm of chromosome 11 (11q23) are the most followed by t(8;21), t(3;21), t(16;21), t(17;21), inv(16), t(8;16) and t(9;22) [4]. DNA topoisomerase II inhibitor-related t-AML most commonly presents with M4 or M5 morphological type and is not associated with MDS. We present a case with M5 morphological type. Pedersen-Bjergaard et al*. *were the first to report the presence of inv(16)(p13q22) as a single chromosomal abnormality in t-AML treated with radiotherapy and subsequently with MOPP (nitrogen mustard, vincristine, procarbazine, and prednisone) in a patient with Hodgkin's disease [15]. In our patient with AC chemotherapy-related AML, inv(16)(p13q22) was one of the three chromosomal abnormalities observed.

In the National Surgical Adjuvant Breast and Bowel Project (NSABP) B25 trial of adjuvant AC chemotherapy for breast cancer, six out of 2534 cases in the high-dose cyclophosphamide/high-dose doxorubicin treatment arm developed M4 or M5 AML, with three patients exhibiting 11q23 abnormalities. Topoisomerase II was accounted for development of leukemia in these patients. Ten other patients developed AML or MDS, with three patients exhibiting M1 or M2 morphology and five patients showing typical alkylating-agent-induced abnormalities of chromosomes 5 or 7 [20]. To the best of our knowledge, our patient's case is the first case of t-AML showing M5 morphology with del 7q, 20q and the inv(16) signal developed three years after chemoradiotherapy for breast cancer.

With recent developments and improved cure rates in cancer therapy, t-MN is an increasingly recognized obstacle. Patients must be informed about the potential risk of developing t-MN following chemotherapy.

## Consent

Written informed consent was obtained from the patient for publication of this case report and any accompanying images. A copy of the written consent is available for review by the Editor-in-Chief of this journal.

## Competing interests

The authors declare that they have no competing interests.

## Authors' contributions

YI analyzed the data and wrote the paper. HF revised the paper and was a contributor to writing the paper. OE helped in collection of data from our patient. PS performed the cytogenetic analysis. AM revised the paper. All authors read and approved the final manuscript.
